# Supplementary material for: Multi‐dataset identification of innovative feature genes and molecular mechanisms in keratoconus
Source: J Cell Mol Med. 2024 Sep 19;28(18):e70079. doi: 10.1111/jcmm.70079 (PMC11412914; doi:10.1111/jcmm.70079)
Supplement: Supplementary file 2 — Material S2. [file JCMM-28-e70079-s002.zip › 7.GSEA analysis/6.SNORA74B/KEGG_GLYCOLYSIS_GLUCONEOGENESIS.html]

Details for gene set KEGG\_GLYCOLYSIS\_GLUCONEOGENESIS[GSEA]

|  || Dataset | SNORA74B.group.cls#h\_versus\_l |
| Phenotype | group.cls#h\_versus\_l |
| Upregulated in class | l |
| GeneSet | KEGG\_GLYCOLYSIS\_GLUCONEOGENESIS |
| Enrichment Score (ES) | -0.4224292 |
| Normalized Enrichment Score (NES) | -1.5131228 |
| Nominal p-value | 0.029354207 |
| FDR q-value | 0.31673428 |
| FWER p-Value | 0.755 |
Table: GSEA Results Summary

  

Fig 1: Enrichment plot: KEGG\_GLYCOLYSIS\_GLUCONEOGENESIS      
 Profile of the Running ES Score & Positions of GeneSet Members on the Rank Ordered List

  

| SYMBOL | TITLE | RANK IN GENE LIST | RANK METRIC SCORE | RUNNING ES | CORE ENRICHMENT || 1 | ADH1B | na | 4400 | 0.246 | -0.0965 | No |
| 2 | LDHAL6A | na | 4598 | 0.239 | -0.0726 | No |
| 3 | ADH6 | na | 4824 | 0.231 | -0.0505 | No |
| 4 | ADH4 | na | 5073 | 0.223 | -0.0301 | No |
| 5 | FBP1 | na | 9128 | 0.111 | -0.1334 | No |
| 6 | ALDOC | na | 10161 | 0.089 | -0.1521 | No |
| 7 | ACSS1 | na | 10420 | 0.085 | -0.1491 | No |
| 8 | AKR1A1 | na | 10981 | 0.075 | -0.1559 | No |
| 9 | ALDH3B1 | na | 12635 | 0.051 | -0.1972 | No |
| 10 | DLD | na | 12649 | 0.051 | -0.1912 | No |
| 11 | ALDH3A2 | na | 13104 | 0.046 | -0.1987 | No |
| 12 | PFKP | na | 13672 | 0.040 | -0.2101 | No |
| 13 | PDHA2 | na | 13714 | 0.039 | -0.2065 | No |
| 14 | ADH7 | na | 15231 | 0.022 | -0.2474 | No |
| 15 | ENO1 | na | 15810 | 0.017 | -0.2621 | No |
| 16 | PDHB | na | 16643 | 0.008 | -0.2851 | No |
| 17 | ACSS2 | na | 16817 | 0.006 | -0.2893 | No |
| 18 | PDHA1 | na | 18249 | -0.009 | -0.3295 | No |
| 19 | ENO3 | na | 18355 | -0.010 | -0.3312 | No |
| 20 | BPGM | na | 18679 | -0.014 | -0.3389 | No |
| 21 | PGM2 | na | 18742 | -0.014 | -0.3389 | No |
| 22 | PFKL | na | 18924 | -0.016 | -0.3421 | No |
| 23 | ALDH7A1 | na | 19848 | -0.027 | -0.3654 | No |
| 24 | GAPDH | na | 20007 | -0.028 | -0.3665 | No |
| 25 | PGAM4 | na | 20127 | -0.030 | -0.3662 | No |
| 26 | LDHC | na | 20197 | -0.030 | -0.3645 | No |
| 27 | TPI1 | na | 20621 | -0.035 | -0.3723 | No |
| 28 | LDHB | na | 21112 | -0.041 | -0.3813 | No |
| 29 | ALDOA | na | 21726 | -0.048 | -0.3930 | No |
| 30 | PGAM1 | na | 22747 | -0.061 | -0.4148 | Yes |
| 31 | HK1 | na | 22849 | -0.063 | -0.4100 | Yes |
| 32 | PFKM | na | 22996 | -0.065 | -0.4061 | Yes |
| 33 | G6PC2 | na | 23090 | -0.067 | -0.4005 | Yes |
| 34 | PGK1 | na | 23700 | -0.076 | -0.4087 | Yes |
| 35 | ALDH3A1 | na | 24095 | -0.082 | -0.4099 | Yes |
| 36 | PKM | na | 24276 | -0.085 | -0.4045 | Yes |
| 37 | LDHA | na | 24376 | -0.087 | -0.3966 | Yes |
| 38 | DLAT | na | 24417 | -0.088 | -0.3869 | Yes |
| 39 | PKLR | na | 24696 | -0.093 | -0.3834 | Yes |
| 40 | HK3 | na | 24705 | -0.093 | -0.3721 | Yes |
| 41 | PCK1 | na | 25015 | -0.099 | -0.3689 | Yes |
| 42 | GPI | na | 25260 | -0.103 | -0.3632 | Yes |
| 43 | ALDH9A1 | na | 25479 | -0.107 | -0.3562 | Yes |
| 44 | ADH1A | na | 25803 | -0.113 | -0.3516 | Yes |
| 45 | HK2 | na | 25913 | -0.115 | -0.3405 | Yes |
| 46 | ENO2 | na | 26364 | -0.124 | -0.3381 | Yes |
| 47 | PGM1 | na | 26743 | -0.134 | -0.3324 | Yes |
| 48 | LDHAL6B | na | 26958 | -0.139 | -0.3215 | Yes |
| 49 | ADH5 | na | 26984 | -0.139 | -0.3050 | Yes |
| 50 | ALDH1B1 | na | 27039 | -0.140 | -0.2892 | Yes |
| 51 | ALDH3B2 | na | 27617 | -0.154 | -0.2867 | Yes |
| 52 | FBP2 | na | 30438 | -0.243 | -0.3380 | Yes |
| 53 | PGAM2 | na | 31034 | -0.270 | -0.3218 | Yes |
| 54 | GALM | na | 31259 | -0.281 | -0.2935 | Yes |
| 55 | ALDOB | na | 31356 | -0.286 | -0.2609 | Yes |
| 56 | GCK | na | 31556 | -0.298 | -0.2297 | Yes |
| 57 | PCK2 | na | 32780 | -0.374 | -0.2187 | Yes |
| 58 | ALDH1A3 | na | 32981 | -0.391 | -0.1760 | Yes |
| 59 | ADH1C | na | 34198 | -0.581 | -0.1392 | Yes |
| 60 | PGK2 | na | 34298 | -0.613 | -0.0663 | Yes |
| 61 | ALDH2 | na | 34359 | -0.636 | 0.0106 | Yes |
Table: GSEA details [plain text format]

  

Fig 2: KEGG\_GLYCOLYSIS\_GLUCONEOGENESIS      
 Blue-Pink O' Gram in the Space of the Analyzed GeneSet

  

Fig 3: KEGG\_GLYCOLYSIS\_GLUCONEOGENESIS: Random ES distribution      
 Gene set null distribution of ES for **KEGG\_GLYCOLYSIS\_GLUCONEOGENESIS**
